# Massively distended, necrotic and hemorrhagic gallbladder in a long-term octreotide-treated patient with added everolimus

**DOI:** 10.1016/j.ijscr.2019.07.014

**Published:** 2019-07-19

**Authors:** Eric Bergeron, Michael Bensoussan

**Affiliations:** Departments of Surgery and Gastroenterology, Charles-LeMoyne Hospital, Greenfield Park, Canada

**Keywords:** Cholelithiasis, Cholecystitis, Carcinoid cancer, Octreotide, Everolimus

## Abstract

**Introduction:**

Long-term treatment with somatostatin analogs, such as octreotide, is well known to promote gallstones formation. Immunosuppressive therapy in renal transplantation is also associated with increased occurrence of gallstones. But acute cholecystitis develops only in a few cholelithiasis patients. However, it is not known whether long-term somatostatin analog therapy and immunosuppressants aggravate the severity of disease if the patient develops cholecystitis.

**Case presentation:**

We present a case of severe cholecystitis in a patient with metastatic carcinoid cancer on octreotide long-acting release therapy for seven years with newly added immunosuppressant, everolimus.

**Discussion:**

Cholelithiasis as well as cholecystitis develop more often in patients on somatostatin analogs and immunosuppressants than in general population. However, morbidity remains negligible.

**Conclusion:**

No conclusion can be drawn on the contribution of somatostatin analogs and immunosuppressant in the occurrence of severe cholecystitis. Prophylactic cholecystectomy is not indicated in patients with this medication.

## Highlights

• Somatostatin analogs and immunosuppressants promote gallstones formation.
• Severe cholecystitis can develop during long-term treatment of metastatic carcinoid cancer.
• There is no proof that severe cholecystitis is caused by somatostatin analogs and immunosuppressants.

## 1 Introduction

The prevalence of biliary stones varies from 10% to 25% in general population [[1], [2], [3], [4], [5]]. The incidence of gallstones increases between 35% to 63% in patients treated with somatostatin analogs for a long time [[6], [7], [8], [9]].

Majority of the patients with somatostatin analogs remain asymptomatic [1,6,10]. In symptomatic patients, the reported cholecystectomy rates vary from 6% to 23% [1,6,7,9,11]. The occurrence of complications, the majority being acute cholecystitis, is slightly increased compared with the general population [6], even though morbidity remains negligible [7].

There have been no reports indicating if the gallstone-related disease in patients on long-term somatostatin analog therapy can be more severe. We present a case of common bile duct stone and severe cholecystitis in a patient with a seven-year history of metastatic carcinoid cancer on octreotide long-acting release (LAR) therapy with recently added immunosuppressive therapy. This interesting case is discussed in light of the recent literature with the necessary recommendations for clinical managing such cases. This work has been reported in line with the SCARE criteria [12].

## 2 Case presentation

In August 2018, a thin, 63 year-old Caucasian female presented to the emergency room with a three-day history of right upper quadrant pain. She was known to have metastatic carcinoid cancer for seven years, receiving treatment with octreotide LAR, which was increased to 50 mg every month one year ago. Everolimus, 10 mg daily, was also added at the same time. During the last year, she developed secondary diabetes which was treated with an oral hypoglycemic agent, metformin.

The patient’s a body temperature was 37.1 °C. She was slightly jaundiced. Murphy’s sign was positive, and a mass was felt in the right upper quadrant. White cell count was 10,400/mm³ with a neutrophil count of 79%. Total bilirubin value was 36 μmol/L (normal: 3–21 μmol/L); aspartate aminotransferase 197 U/L (normal: 5–35 U/L) and alanine aminotransferase 175 U/L (normal: 5–35 U/L). Blood glucose level was 9.6 mmol/L (normal: 4.0–6.0 mmol/L) with HbA1c of 0.078 (normal: 0.045-0.060). Four months before, chromogranin A was 374 μg/L (normal: 0–82 μg/L).

**Fig. 1:**
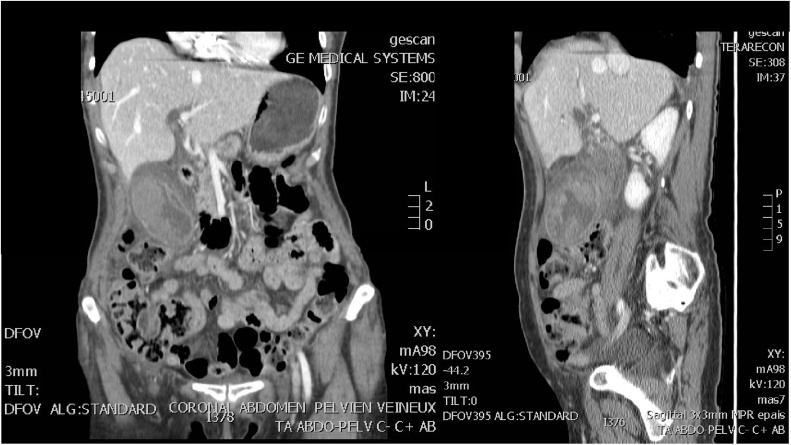
Coronal and sagittal enhanced CT images showing a markedly distended and wall-thickened gallbladder.

Abdominal ultrasound showed a massively distended gallbladder with multiple gallstones. The common bile duct was dilated as were the intrahepatic bile ducts. Computed tomography (Fig. 1) demonstrated gallbladder with a thickened wall, measuring 15 cm in length and 5 cm in width. Common bile duct was measured at 1.6 cm with a possibility of stricture.

**Fig. 2:**
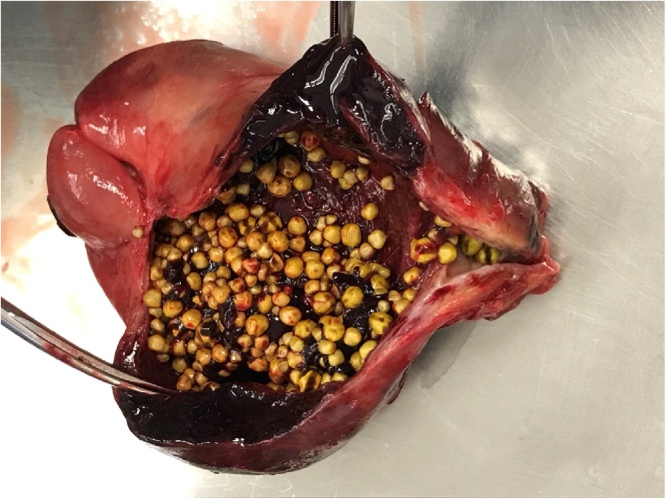
Gallbladder showing multiple cholelithiasis, thickened and necrotic wall with hemorrhage.

The patient was admitted with a diagnosis of cholecystitis and treatment with piperacillin-tazobactam was initiated. An endoscopic retrograde cholangiopancreatography was scheduled to rule out a stricture or the presence of stones, and this test was done four days later. One obstructive stone was retrieved and there was no evidence of underlying stricture. The same day, an open cholecystectomy was carried out. Laparotomy was elected because of the large size of the gallbladder in a thin patient. At laparotomy, there was no evidence of intraperitoneal tumor. The gallbladder was removed without complication. A drain was left in place. Postoperative care was uneventful. The gallbladder contained several stones and had a necrotic and hemorrhagic wall (Fig. 2).

After reviewing the chart of the patient, an abdominal tomography had been performed once a year from the initial diagnosis. The patient was diagnosed in 2011 with an ovarian carcinoid tumor. She underwent bilateral salpingo-oophorectomy, hysterectomy and pelvic lymph node dissection. Following surgery, treatment with octreotide LAR was initiated. She developed metastases in nodes, bones and muscles with few months. She developed hearth complications and had tricuspid valve replacement and pacemaker installation in 2013. Everolimus was added to her treatment in August 2017, one year prior to her presentation to the emergency room in 2018.

All exams until 2017 showed a normal nondistended gallbladder. Prior to the acute presentation in 2018 when multiple stones were seen in the gallbladder, no stones were demonstrated; however, records showed that there were no ultrasound exams.

## 3 Discussion

The effect of somatostatin analogs on gallbladder and secondary development of gallstones is well known [7,13]. They abolish the release of cholecystokinin from the small intestine, the contractile response of the gallbladder to cholecystokinin, the post prandial relaxation of the sphincter of Oddi, and the mobility and emptying of the gallbladder. Moreover, these drugs induce lithogenic changes in bile composition leading to stone formation [7,9].

The incidence of gallstones in somatostatinoma is 59% for patients with pancreatic tumors and 27% in patients with intestinal tumors [14]. Nearly 63% of the patients on somatostatin analogs develop gallstones [6], an incidence higher than the general population [[1], [2], [3], [4], [5]].

Most studies have been conducted in patients affected by acromegaly [1,7,9,10]. In two recent trials in patients with metastatic neuroendocrine tumors receiving somatostatin analogs octreotide LAR [15] and lanreotide [16], the development of gallstones was 10% and 14%, respectively, and this was considered as a drug-related adverse event.

Development of stones occurred after an average period of 3 years [1,7,9], and increases with the dose of medication [9] and duration of treatment [7]. The risk to undergo cholecystectomy for biliary colic or complications of gallstones varies from 11% to 23% [1,6,9]. The cumulative 5-year incidence of acute gallbladder disease (cholecystitis, pancreatitis, cholangitis) is reported to be 8.1% [6]. This same study also reported a 5-year cumulative risk of cholecystectomy in patients receiving somatostatin analogs for carcinoid cancer to be 19% compared to 2.3% in untreated patients [6]. In the general population, patients with gallstones develop symptoms at a rate of 1 to 3% per year, a 20% incidence of symptoms within 20 years and 10% of symptomatic patients having cholecystitis [5].

In the case we present here, cholecystitis was severe. There are no previous reports indicating more severe disease in case of complications due to gallbladder disease, but negligible morbidity is reported [6,7]. The concomitant diabetes and treatment with immunosuppressive therapy, may have contributed. However, there is only one case of cholecystitis with everolimus [17]. Moreover, everolimus is independently associated with a diminished hazard of developing new onset of cholelithiasis after renal transplant [18]. No relationship between newly added everolimus and the occurrence of cholecystitis, even severe, may thus be established.

Distension of the gallbladder was not present in previous follow-up. Massive distension at the time of presentation is certainly due to the effect of octreotide on the gallbladder combined with the occlusion caused by the stones. However, it is not possible to know precisely when the gallstones appeared since only tomography, which is less sensitive to detect stones than ultrasound, has been done for follow-up. We also cannot conclude, neither from this interesting case nor from the literature, that cholecystitis is more severe in patients on somatostatin analogs either alone or in combination with immunosuppressive therapy [11,18].

During the management of the present case, we had to wait for the results of the endoscopic retrograde pancreatography to rule out a pathologic process on the common bile duct (stricture, impacted stones) that could have influenced the operative approach to this problem, at the same time as cholecystectomy. We elected to perform open cholecystectomy because of the massive distension of the gallbladder, which could impair good visualisation of the anatomy in this thin patient. However, laparoscopic cholecystectomy usually can be done securely in patients with cholecystitis [1], but several considerations necessitate conducting open cholecystectomy in patients who have been previously operated for abdominal carcinoid tumors [7].

Some authors advocate prophylactic cholecystectomy if a laparotomy is planned, even for gallbladder with no stones [6]. However, we do not support prophylactic cholecystectomy if there are no gallstones at the time of operation, in agreement with other studies [11]. A recent retrospective observational study on patients with neuroendocrine tumors receiving somatostatin analogs [1], it was reported that 85% of the patients who were known to have gallstones remained asymptomatic. The same study also reported that 71% of those patients, who newly developed gallstones, also remained asymptomatic [1]. Even if the incidence of gallstones is high during the treatment with somatostatin analog, their occurrence may be years later [1,7,9]. Majority of the patients may remain asymptomatic up to 20 years [1,10,11] with low cumulative incidence of complications due to gallbladder disease [6,7,9], and with minimal morbidity [7]. For these reasons, prophylactic cholecystectomy is not indicated in patients who have gallstones at the initiation of somatostatin analogs [11] nor is any monitoring, as suggested by others [7,9], for the occurrence of gallstones while on treatment.

## 4 Conclusions

The incidence of gallstones is high in patients on somatostatin analogs. No conclusion can be drawn on the contribution of everolimus in the occurrence of gallstones and cholecystitis. The development of symptoms in patients on somatostatin analogs who develop gallstones is increased but remain low in comparison to general population. The risk of complications of gallstones remains low, the disease is rarely severe, and the morbidity of surgical treatment of gallbladder disease is minimal.

In the presence of gallstones, either at the initiation or during ongoing treatment with somatostatin, cholecystectomy is not indicated. Cholecystectomy is indicated in patients with gallstones-related symptoms or complications. Prophylactic cholecystectomy is indicated during laparotomy for abdominal tumor in the presence but not in the absence of gallstones.

## Funding

EB will pay for the submission.

## Ethical Approval

Ethical approval has been exempted by our institution.

## Consent

Written informed consent has been obtained from the patient.

In this paper, and in the images, there is no possibility to identify the patient.

## Author contribution

EB revised the record of the patient.

EB and MB reviewed the literature, wrote the paper, et revised the manuscript.

## Registration of Research Studies

W/O.

## Guarantor

EB accept the responsibility for this work.

## Provenance and peer review

Not commissioned, externally peer-reviewed.

## Declaration of Competing Interest

None.
